# Perianal fistulizing Crohn's disease and overall risk of cancer: No red flag

**DOI:** 10.1002/ueg2.12401

**Published:** 2023-05-04

**Authors:** Paul McLellan, Julien Kirchgesner

**Affiliations:** ^1^ Department of Gastroenterology AP‐HP, Hôpital Saint‐Antoine Paris France; ^2^ Department of Gastroenterology Sorbonne Université INSERM Institut Pierre Louis d’Epidémiologie et de Santé Publique AP‐HP, Hôpital Saint‐Antoine Paris France

Inflammatory bowel disease (IBD) is not only associated with an increased risk of colorectal cancer, with the greatest risk in patients with extensive and chronically active disease, but also with an increased risk of skin cancer, haematological malignancies, and urinary tract cancer.^1^ Perianal fistulizing disease affects 20%–30% of patients with Crohn's disease (CD)^2^ and has been associated with an increased risk of anal cancer.^3^ Despite advances in the therapeutic management, the risk of poor disease course remains substantial.^4^ Chronic perianal inflammation plays a key role in carcinogenesis and due to their location, cancers in this setting are rarely diagnosed at an early stage.^5^ While immunosuppressive treatments contribute to the increased risk of cancer in patients with IBD,^6^ patients with perianal fistulizing CD are more exposed to immunosuppressive treatments and for a longer period of time compared to the overall population of patients with IBD.^7^ Finally, the overall risk of cancer in patients with perianal fistulizing CD remained to be elucidated.

In the current issue of the *United European Gastroenterology Journal*, Podmore et al. assessed the risk of cancer in patients with perianal fistulizing CD based on German administrative healthcare databases including 4.8 million persons.

The authors identified 824 patients with perianal fistulizing CD among 10,208 patients with CD. Patients were followed up from January 2015 to December 2020, accounting for 4222 person‐years of follow‐up in patients with perianal fistulizing CD. The authors observed that the incidence of any type of cancer was higher in patients with non‐perianal fistulizing CD compared with patients with perianal fistulizing CD (2365 [95% CI 2219–2519] and 1184 [95% CI 879–1561] cancers per 100,000 person years, respectively). Compared to the general population, the standardized incidence ratio of any type of cancer in patients with perianal fistulizing CD was more than 1.5‐times higher, which is in line with findings observed in the overall population of patients with CD. Of note, the prevalence of anal and perianal cancer was higher in patients with perianal fistulizing CD (1.3%) compared with non‐perianal fistulizing CD (0.6%).

Some limitations need to be acknowledged. Treatment exposure was assessed only at cohort entry, which could lead to treatment misclassification. Smoking status was not collected and residual confounding could not be excluded. The study was also underpowered to specifically assess the risk of incident anal cancer in patients with perianal fistulizing CD. Nevertheless, this study is of great value compared to the available literature, notably by being the first study reporting the overall risk of cancer in patients with perianal fistulizing CD.

Overall, these reassuring data suggest no overall increased risk of cancer in patients with perianal fistulizing CD compared with patients with non‐perianal fistulizing CD. The risk of perianal cancer remains to be tackled in patients with perianal fistulizing CD. While waiting for the development of dedicated surveillance programs, long lasting anal fistulas, especially in case of persistent pain, should raise attention and discuss clinical examination with histological samples under general anesthesia.

## Data Availability

There are no original data included.
